# Invasive *Ureaplasma* infection in a pediatric patient: a case report

**DOI:** 10.1128/asmcr.00164-25

**Published:** 2026-02-12

**Authors:** Brice Laney, Daniel E. Dulek, David C. Gaston, Natalya Beneschott, Sophie E. Katz, Jessica Gillon, Stephanie Rolsma

**Affiliations:** 1 Department of Pharmacy, Monroe Carell Junior Children’s Hospital at Vanderbilt 21629 , Nashville, Tennessee, USA; 2 Division of Pediatric Infectious Diseases, Department of Pediatrics, Vanderbilt University Medical Center 12328, Nashville, Tennessee, USA; 3 Department of Pathology, Microbiology, and Immunology, Vanderbilt University Medical Center 12328, Nashville, Tennessee, USA; 4 Vanderbilt Vaccine Research Program, Department of Pediatrics, Vanderbilt University Medical Center 12328, Nashville, Tennessee, USA; Pattern Bioscience, Austin, Texas, USA

**Keywords:** pediatrics, pediatric, *Ureaplasma*, rituximab, levofloxacin, doxycycline, obinutuzumab, *Ureaplasma *infections, *Ureaplasma urealyticum*

## Abstract

**Background:**

*Ureaplasma* spp. are a rare cause of invasive disease outside of the neonatal period. Recent studies have shown that immunosuppression with B-cell depleting therapies, such as rituximab or obinutuzumab, can increase the risk of invasive infection. We present a case in which a patient receiving B-cell depleting therapy for anti-neutrophil cytoplasmic antibody-associated vasculitis and pauci-immune nephritis was diagnosed with disseminated, invasive *Ureaplasma* infection.

**Case Summary:**

A 17-year-old female with documented B-cell depletion presented to the emergency department from the pediatric infectious disease clinic with persistent shoulder pain. Within the first month of admission, the patient developed multifocal abscesses with joint involvement and an echodensity resembling a possible vegetation, all of which were unresponsive to broad-spectrum antibiotics. A broad-spectrum polymerase chain reaction test detected *Ureaplasma urealyticum* in aspirated joint fluid from multiple anatomic sites. Clinical improvement occurred after initiation of combination therapy with intravenous doxycycline and levofloxacin.

**Conclusion:**

We describe a patient with documented iatrogenic B cell depletion presenting with a disseminated, multifocal invasive *Ureaplasma* infection including osteoarthritis, cellulitis, and soft tissue abscess. Following a 6-week course of levofloxacin, the patient’s C-reactive protein, fever, and pain resolved without further evidence of disease. This case report highlights the importance of considering disseminated, invasive *Ureaplasma* spp. when evaluating B-cell depleted patients with challenging multifocal symptom complexes. In addition, this case report highlights the critical role of molecular diagnosis in identifying this fastidious pathogen.

## INTRODUCTION

*Ureaplasma urealyticum* and *Ureaplasma parvum* are common colonizers of the lower genital tract of sexually active adults and have also been isolated from the neonatal respiratory tract (1). In addition, *Ureaplasma* causes invasive neonatal infections, chorioamnionitis, urethritis, surgical infections, and post-lung transplant hyperammonemia syndrome (1). Less common presentations of acute invasive infection, including osteomyelitis, non-neonatal pneumonia, pericarditis, endocarditis, and meningitis, have been reported over the last two decades (2–4). Importantly, several prior reports have identified humoral immune deficiency to be a key risk factor in severe invasive infections with mollicute*s,* which include *Ureaplasma and Mycoplasma* spp. (3, 5).

Here, we present a case of invasive *Ureaplasma* infection in a patient who had previously received B-cell depleting therapy with obinutuzumab. The diagnosis was made by broad-range 16S rRNA PCR and confirmed by targeted PCR testing. To our knowledge, this is the first report of an invasive *Ureaplasma* infection linked to the utilization of obinutuzumab.

## CASE PRESENTATION

A 17-year-old female presented to a tertiary, academic medical center in Nashville, Tennessee, with new-onset, persistent shoulder pain. Her medical history was significant for chronic kidney disease due to anti-neutrophil cytoplasmic antibody (ANCA)-associated vasculitis and pauci-immune nephritis managed with B-cell depleting therapy and requiring intermittent hemodialysis. B-cell depleting therapy was initiated approximately 4 years prior to presentation with initial treatment with rituximab. She experienced anaphylaxis with rituximab infusions and underwent a brief switch to ofatumumab, receiving four doses approximately 2 years prior to the current presentation. This was followed by long-term immunosuppression with obinutuzumab, administered every 6 months, which was initiated 14 months prior to the current presentation. The most recent B-cell-depleting therapy with obinutuzumab was received 7 months prior to presentation. Subsequent doses were deferred due to persistent B-cell depletion that extended to her acute presentation (peripheral blood CD19+ lymphocyte count by flow cytometry was undetectable 4 months prior to and 14 days following the present hospitalization). Additionally, the patient had recent admissions for a left renal abscess and a culture-negative site infection of her right intrajugular hemodialysis catheter. The former was treated with an empiric 2-month course of ciprofloxacin leading up to the present admission. The latter required removal and replacement of her hemodialysis catheter in addition to intravenous (IV) vancomycin for 6 days followed by oral clindamycin for a 2-week course of antibiotics.

Initial X-ray imaging of the shoulder was interpreted as normal, and the patient was admitted for pain management and further evaluation. On day 2 of admission, the patient had onset of fever to 102.7°F and an elevated C-reactive protein (CRP) of 147 mg/L (range, 0.1–1.7 mg/L), prompting an orthopedics consultation and further imaging with a transthoracic echocardiogram (TTE) and magnetic resonance imaging (MRI). Despite negative imaging, the patient developed progressive shoulder pain, contralateral facial swelling, and pain of the right temporomandibular joint (TMJ). Given her history of poor dentition and potential facial abscess, she was started on empiric IV ceftriaxone 2,000 mg every 24 h and IV clindamycin 600 mg every 8 h on day 8 of admission. On day 10 of hospitalization, computed tomography (CT) scans of the neck, chest, abdomen, and pelvis identified a right TMJ abscess, right-sided facial cellulitis, potential septic arthritis in the left sternoclavicular joint, and cellulitis or tissue ulceration in the left gluteal region. These findings prompted surgical drainage and cultures of the masticator space abscess and sternoclavicular joint on day 11. Aspirated fluid from both sites was sent for bacterial, anaerobic, and fungal cultures. Fluid from the masticator space abscess was also sent for acid-fast bacillus (AFB) culture. All cultures from these sites ultimately revealed no growth.

On day 14 of admission, serum IgG level was 969 mg/dL (range, 600–1,310 mg/dL) and flow cytometry of peripheral blood demonstrated normal CD3, CD4, and CD8 lymphocyte counts with B cells below the lower limit of quantification. On day 16 of admission, an ultrasound of the left gluteal region revealed a small abscess requiring drainage. Fluid from the gluteal abscess was sent for bacterial, AFB, anaerobic, and fungal cultures. Given the clinical concern for culture-negative multifocal abscesses, the remaining serous fluid from the previously drained TMJ abscess from day 11 of admission was sent for broad-range 16S rRNA PCR at the University of Washington on day 17. Following drainage, the patient’s CRP declined and prompted a transition to oral amoxicillin and clindamycin on day 21. By day 25 of admission, the patient’s CRP began to rise, prompting re-initiation of IV ceftriaxone, initiation of IV metronidazole, and discontinuation of clindamycin and amoxicillin. On day 27, the patient’s clinical condition worsened with an expansion of pain to the left hip, groin, and knee. MRI findings included pelvic myositis, a left hip effusion, and a left knee effusion prompting the addition of IV vancomycin. Joint aspirates each had several thousand total nucleated cells of neutrophil predominance and relatively few red blood cells.

Her clinical condition continued to evolve with the development of chest pain and persistently elevated CRP, prompting a repeat TTE on hospital day 28. The TTE revealed a new echodensity attached to the tricuspid valve concerning for a thrombus or vegetation. On that same day, the broad-range 16S rRNA PCR (University of Washington Molecular Microbiology lab) of previously aspirated TMJ fluid resulted in detection of *Ureaplasma urealyticum*. To that point, all previous blood cultures and testing had been negative for a causative infectious pathogen (Table 1). Urine and aspirates of the knee, hip, and TMJ were sent for PCR identification, culture, and susceptibility testing at the University of Alabama at Birmingham’s (UAB) Diagnostic Mycoplasma Laboratory. (6) *Ureaplasma urealyticum* was detected and isolated in culture for all samples.

**TABLE 1:** Patient cultures and diagnostic timeline

Date	Location	Testing performed	Results^*a*^
Day 2	Blood (peripheral)	Aerobic	No growth
Day 2	Blood (central)	Aerobic	No growth
Day 4	Urine	Urine culture	No growth
Day 7	Blood (central)	Aerobic	No growth
Day 7	Right cheek wound	Aerobic, anaerobic	*Candida albicans*
Day 8	Blood (peripheral)	Aerobic, anaerobic	No growth
Day 11	Sternoclavicular abscess	Anaerobic, acid-fast bacilli, fungal	No growth
Day 11	TMJ abscess	Aerobic, anaerobic, fungal, broad-range 16S rRNA PCR with sequencing	**Broad-range 16S rRNA PCR with sequencing: *Ureaplasma urealyticum***
Day 16	Gluteal abscess	Aerobic, anaerobic, acid-fast bacilli, fungal	No growth
Day 23	Left knee synovial fluid	Aerobic, anaerobic, fungal	No growth
Day 26	Blood (peripheral)	Aerobic	No growth
Day 28	Blood (central)	Aerobic	No growth
Day 28	Blood (central)	Aerobic	No growth
Day 28	Blood (peripheral)	Aerobic	No growth
Day 29	Left hip synovial fluid	Aerobic, anaerobic, *Ureaplasma* PCR and culture	***Ureaplasma* PCR and culture positive: *Ureaplasma urealyticum***
Day 29	Left knee synovial fluid	Aerobic, anaerobic, acid-fast bacilli, fungal, *Ureaplasma* PCR and culture	***Ureaplasma* PCR and culture positive: *Ureaplasma urealyticum***
Day 29	TMJ abscess	Aerobic, anaerobic, acid-fast bacilli, fungal, *Ureaplasma* PCR and culture	***Ureaplasma* PCR and culture positive: *Ureaplasma urealyticum***
Day 29	Urine	*Ureaplasma* PCR and culture	***Ureaplasma* PCR and culture positive: *Ureaplasma urealyticum***
Day 40	Left hip synovial fluid	*Ureaplasma* PCR and culture	Negative

^*a*^ Bold results indicate clinically significant findings.

At the time of *Ureaplasma urealyticum* identification, dual coverage with IV doxycycline 100 mg every 12 h and IV levofloxacin 500 mg every 24 h was started until susceptibilities could be obtained. Vancomycin and metronidazole were discontinued, but ceftriaxone was continued to ensure clinical improvement given concern for culture-negative endocarditis possibly due to a second pathogen. On hospital day 33, ceftriaxone was discontinued. By day 37, the patient was converted to oral therapy with doxycycline and levofloxacin. On day 40, susceptibilities returned revealing the isolate susceptible to erythromycin (0.25 mcg/mL), tetracycline (0.125 mcg/mL), and moxifloxacin (2 mcg/mL). Based on the results, the patient’s regimen was narrowed to oral levofloxacin monotherapy.

**Fig 1:**
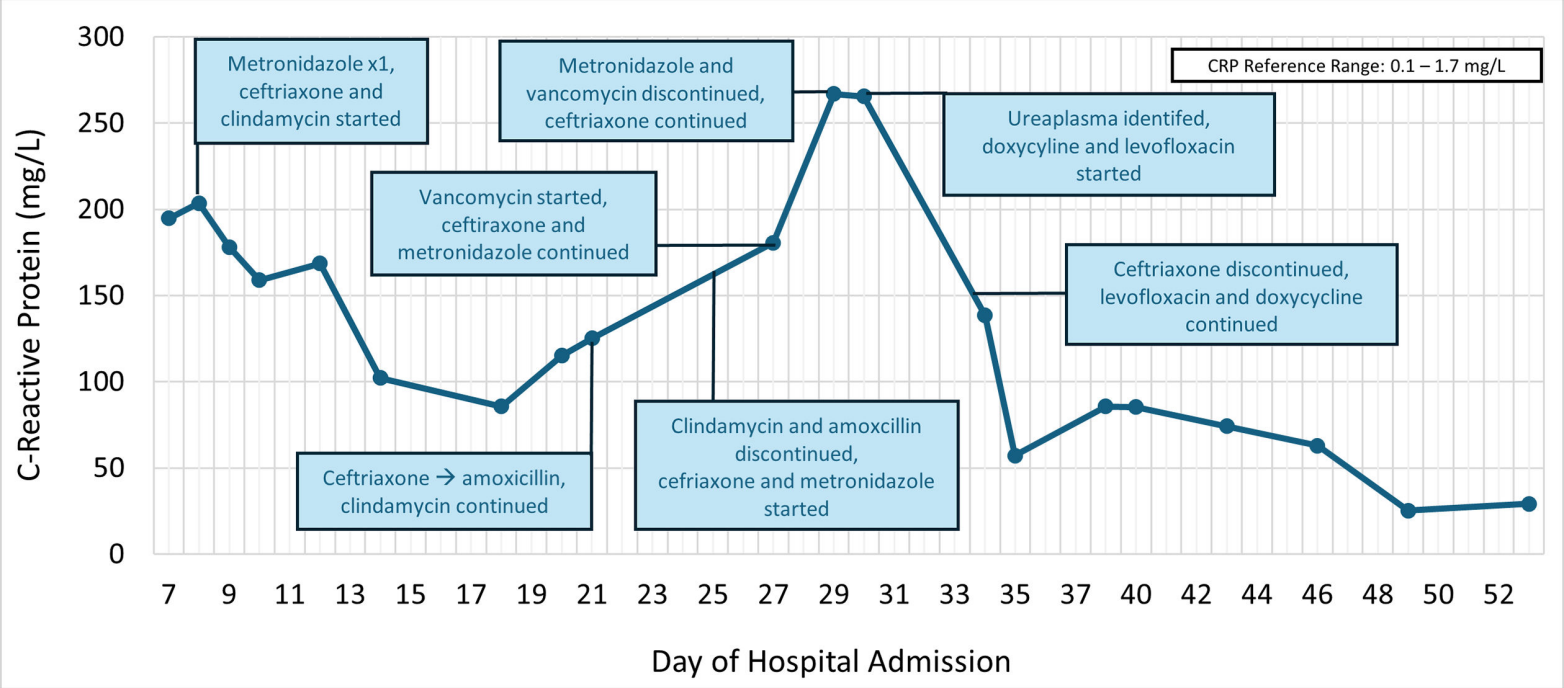
C-reactive protein and antibiotic therapies during hospital admission.

By day 44, the patient’s CRP had decreased to 138.5 mg/L from a peak of 266.9 mg/L on hospital day 29 (Fig. 1). On day 53 of admission, a repeat TTE found no evidence of the echodensity that was previously noted. The patient was discharged home on day 57 with a planned 12-week duration of levofloxacin therapy starting from the date of last surgical intervention—hospital day 40. At the outpatient follow-up, the patient and caregiver noted improvement in strength with resolution of joint pain and swelling. Due to documented variable adherence, the course of levofloxacin was extended for an additional 3 weeks, and she completed a total duration of 4 months of antibiotic therapy.

## DISCUSSION

*Ureaplasma* spp. are fastidious bacteria that commonly colonize the genital tract of sexually active adults and the throat, eyes, umbilicus, and perineum of newborn infants. Manifestations of *Ureaplasma* infection in pediatrics most frequently present as neonatal pneumonia or bacteremia (1). Immunocompromised patients are also at increased risk of invasive disease related to *Ureaplasma*, which may present as osteomyelitis, pneumonia, pericarditis, meningitis, and progressive sinopulmonary disease (1). Our patient’s presentation of multifocal arthritis and soft tissue abscesses with possible endocarditis without hypogammaglobulinemia represents an uncommon manifestation of invasive *Ureaplasma* infection.

Anti-CD20 monoclonal antibodies are commonly used for autoimmune and malignant processes. These agents have known associations with serious infections secondary to prolonged B-cell depletion and hypogammaglobulinemia. An increasing number of cases of invasive *Ureaplasma* in both adult and pediatric patients have been reported (2–4). A 2019 article revealed that approximately 71% of invasive *Ureaplasma* infections occurred in patients with documented hypogammaglobulinemia or receipt of rituximab, an anti-CD20 monoclonal antibody (3). Since 2019, a growing body of literature suggests a link between invasive disease in patients who were B-cell depleted following receipt of anti-CD20 monoclonal antibodies (3, 7–9). These cases include a teenage patient with *Ureaplasma parvum* meningitis (2).

*Ureaplasma* is difficult to identify microbiologically as it is not visible on gram stain and does not grow on typical media (3). Instead, it requires the use of Shepards 10B broth incubated at 37°C, or A8 sodium media incubated in an atmosphere of room air with supplemental CO_2_ or in an environment of 95% N_2_ plus supplemental CO_2_. These media allow for identification within 2 to 4 days (10). At our institution, we utilize BacT/ALERT (bioMérieux) blood cultures with continuous monitoring for aerobic and anaerobic species, and BACTEC (Becton Dickinson) blood cultures for acid-fast bacilli. These contain the anticoagulant sodium polyanethol sulfonate (SPS) which may inhibit the growth of *Mycoplasma* and *Ureaplasma* spp. preventing them from reaching the threshold of positivity and delaying diagnosis (11–16). However, culture remains important to guide treatment decisions since antibiotic resistance is common (1). Given the patient’s prior admission for right IJ hemodialysis catheter site infection and development of right-sided facial swelling and multifocal other sites of infection, the initial focus of evaluation and management for her was on identifying and treating either typical bacterial pathogens, such as *Staphylococcus aureus* or oropharyngeal anaerobic bacteria. However, after numerous negative blood cultures, a broad-range 16S rRNA PCR was performed at the University of Washington, which ultimately identified *Ureaplasma urealyticum*. 16S rRNA PCR testing is often utilized when an infection is suspected and conventional methods for identification have been exhausted. At this time, real-time PCR assays, such as the one developed by the UAB Diagnostic Mycoplasma Laboratory, are preferred to detect *Ureaplasma* species due to increased sensitivity and rapid turnaround time (6). Once the causative pathogen was identified, synovial fluid samples were sent for dedicated *Ureaplasma* culture and susceptibility testing at UAB’s Diagnostic Mycoplasma Laboratory. The inability to routinely culture and perform susceptibility testing on these organisms illustrates the many challenges that exist in diagnosing and treating invasive *Ureaplasma* infections.

Based on our review, this would be only the third case of possible *Ureaplasma*-related endocarditis reported in literature. The other two cases occurred in immunocompromised adults—one with common variable immunodeficiency and one undergoing treatment for lymphoma (4, 17). The isolate of *Ureaplasma urealyticum* identified in our patient was found to be susceptible to erythromycin (0.25 mcg/mL), tetracycline (0.125 mcg/mL), and moxifloxacin (2 mcg/mL), reflecting a similar susceptibility pattern to those noted in a 2016 report on United States isolates of the organism (18). Based on the available data and reports of resistance, our patient was started on combination therapy with levofloxacin and doxycycline, which was narrowed to levofloxacin monotherapy. On appropriate therapy, our patient’s CRP followed a similar trajectory outlined in other case reports (2, 7, 19).

Numerous studies have identified an association between *Ureaplasma* infection and the development of hyperammonemia syndrome in immunocompromised patients (20). This association was first identified in lung transplant recipients, though has since been reported in other categories of immunocompromising conditions, primarily hematopoietic cell transplantation and receipt of myeloablative chemotherapy (20). The present patient had normal ammonia levels sent during her hospitalization after identification of her Ureaplasma infection. The mechanism underlying why some immunocompromised patients develop hyperammonemia syndrome and others do not remains unclear (20).

We present the first case of an invasive *Ureaplasma* infection linked to the obinutuzumab-induced B-cell depletion and the first report of possible endocarditis caused by an invasive *Ureaplasma* species in a pediatric patient. Identifying the microbiologic etiology for this patient’s presentation was challenging due to the multifocal distribution of symptoms and the atypical locations of her arthritis. Importantly, the first identification of *Ureaplasma* in this patient was made by a broad-range 16S rRNA PCR assay, highlighting the important role that these assays play in making challenging diagnoses. This patient’s clinical course highlights the need for infectious diseases clinicians to have a high index of suspicion of *Ureaplasma* spp. infections in B cell-depleted pediatric patients.

## References

[1] Kimberlin DW, Banerjee R, Barnett E, Lynfield R, Sawyer MH. 2024. *Ureaplasma urealyticum* and *Ureaplasma parvum* infections. In Red Book: 2024–2027 Report of the Committee on Infectious Diseases. American Academy of Pediatrics.

[2] Ehrnström B, Haugan MS, Andreasen JB, Ellingsen A. 2024. Immunocompromised teenager with meningitis caused by Ureaplasma parvum BMJ Case Rep 17:e257261. doi:10.1136/bcr-2023-257261 PMC10921514 38453229

[3] Jhaveri VV, Lasalvia MT. 2019. Invasive Ureaplasma infection in patients receiving rituximab and other humoral immunodeficiencies-a case report and review of the literature. Open Forum Infect Dis 6:ofz399. doi:10.1093/ofid/ofz399 31660361 PMC6790395

[4] Frangogiannis NG, Cate TR. 1998. Endocarditis and Ureaplasma urealyticum osteomyelitis in a hypogammaglobulinemic patient. A case report and review of the literature. J Infect 37:181–184. doi:10.1016/s0163-4453(98)80174-6 9821094

[5] Furr PM, Taylor-Robinson D, Webster ADB. 1994. Mycoplasmas and ureaplasmas in patients with hypogammaglobulinaemia and their role in arthritis: microbiological observations over twenty years. Ann Rheum Dis 53:183–187. doi:10.1136/ard.53.3.183 8154936 PMC1005283

[6] Xiao L, Glass JI, Paralanov V, Yooseph S, Cassell GH, Duffy LB, Waites KB. 2010. Detection and characterization of human Ureaplasma species and serovars by real-time PCR. J Clin Microbiol 48:2715–2723. doi:10.1128/JCM.01877-09 20554828 PMC2916572

[7] Schwartz DJ, Elward A, Storch GA, Rosen DA. 2019. Ureaplasma urealyticum pyelonephritis presenting with progressive dysuria, renal failure, and neurologic symptoms in an immunocompromised patient. Transpl Infect Dis 21:e13032. doi:10.1111/tid.13032 30472777 PMC6542459

[8] Schwartz S, Aldrich A, Kessler E, Abulaban K, Steinke JM. 2023. Disseminated Ureaplasma polyarthritis in a renal transplant recipient. Pediatr Transplant 27:e14538. doi:10.1111/petr.14538 37149734

[9] Harold R, Simon GL, Akselrod H, Siegel MO, Roberts A. 2021. Ureaplasma septic polyarthritis in a young woman with neuromyelitis optica receiving rituximab. BMJ Case Rep 14:e237916. doi:10.1136/bcr-2020-237916 PMC7852972 33526524

[10] Waites KB, Bébéar C. 2023. *Mycoplasma* and *Ureaplasma*, p 1–23. In Carroll KC, Pfaller MA (ed), Manual of Clinical Microbiology, 13th ed. John Wiley & Sons, Inc.

[11] Kelly VN, Garland SM, Gilbert GL. 1987. Isolation of genital mycoplasmas from the blood of neonates and women with pelvic infection using conventional SPS-free blood culture media. Pathology (Phila) 19:277–280. doi:10.3109/00313028709066563 3431914

[12] U.S. Food and Drug Administration. 1998. Summary of safety and effectiveness: BACTEC MYCO/F LYTIC CULTURE MEDIUM

[13] U. S. Food and Drug Administration. 2013. Premarket notification: BacT/ALERT FN Plus Culture Bottle

[14] U.S. Food and Drug Administration. 2013. Premarket notification: BacT/ALERT FA Plus Culture Bottle

[15] U.S. Food and Drug Administration. 2013. Premarket notification: BacT/ALERT PF Plus Culture Bottle

[16] U.S. Food and Drug Administration. 1998. Premarket notification: BACTEC MYCO/F LYTIC CULTURE MEDIUM

[17] Korytny A, Nasser R, Geffen Y, Friedman T, Paul M, Ghanem-Zoubi N. 2017. Ureaplasma parvum causing life-threatening disease in a susceptible patient. BMJ Case Rep 2017:bcr2017220383. doi:10.1136/bcr-2017-220383 PMC5614231 28814589

[18] Fernández J, Karau MJ, Cunningham SA, Greenwood-Quaintance KE, Patel R. 2016. Antimicrobial susceptibility and clonality of clinical Ureaplasma isolates in the United States. Antimicrob Agents Chemother 60:4793–4798. doi:10.1128/AAC.00671-16 27246773 PMC4958210

[19] Newman NA, Pomputius WF, Kurachek S. 2025. Complicated pediatric pneumonia due to Ureaplasma parvum : a case report. Pediatr Infect Dis J 17. doi:10.1097/INF.0000000000005065 41243126

[20] Roberts SC, Malik W, Ison MG. 2022. Hyperammonemia syndrome in immunosuppressed individuals. Curr Opin Infect Dis 35:262–268. doi:10.1097/QCO.0000000000000828 35665721 PMC9179651

